# Activities of wild‐type and variant tissue‐type plasminogen activators retained on vascular endothelial cells

**DOI:** 10.1002/2211-5463.12057

**Published:** 2016-04-01

**Authors:** Yuko Suzuki, Hideto Sano, Martyna Tomczyk, Tomasz Brzoska, Tetsumei Urano

**Affiliations:** ^1^ Department of Medical PhysiologyHamamatsu University School of MedicineJapan

**Keywords:** fibrinolysis, tissue‐type plasminogen activator, vascular endothelial cells

## Abstract

We reported that tissue‐type plasminogen activator (tPA) secreted from vascular endothelial cells (VECs) is retained on the cell surface and effectively evokes both plasminogen activation and fibrin clot dissolution (fibrinolysis) on VECs. Here, to evaluate possibly different behaviors of variants of tPA, we quantitatively assessed these two events separately using green fluorescent protein (GFP)‐conjugated tPA in cultured human VECs. The amount of secreted wild‐type tPA‐GFP correlated well with both the activities of plasminogen activation (*r* = 0.66) and fibrinolysis (*r* = −0.93). A variant of tPA‐GFP, with a lower affinity to the surface of VECs but higher affinity to fibrin, showed higher fibrinolysis and lower plasminogen activation activity compared to the wild‐type.

**Abbreviations**
CLSM: confocal laser scanning microscopy
CLT: clot lysis time
GFP: green fluorescent protein
PAI‐1: plasminogen activator inhibitor‐1
plg‐568: Alexa Fluor 568‐labeled plasminogen
ROI: region of interest
RPG: rate of plasmin generation
RR: retained ratio
TIRF: total internal reflection fluorescence
tPA: tissue‐type plasminogen activator
VECs: vascular endothelial cells

Tissue‐type plasminogen activator (tPA), secreted from vascular endothelial cells (VECs) triggers intravascular fibrinolysis by activating plasminogen to plasmin 1. Using green fluorescent protein (GFP)‐conjugated tPA (tPA‐GFP) and total internal reflection fluorescence (TIRF) microscopy, we studied the unique exocytotic dynamics of tPA‐GFP and observed that tPA‐GFP was retained on the VEC surface in a heavy‐chain‐dependent manner after exocytosis 2. Such a slow discharge of tPA was also reported in adrenal chromaffin cells 3, 4, in which tPA showed lower diffusion mobility after the fusion of the granule membrane with the plasma membrane due to a slower fusion‐pore expansion by tPA.

Unlike other members of the family of serine proteases, tPA is secreted as an active form 5, and thus its ‘retention’ on the solid surface is directly related to the facilitated plasmin generation 6, since its substrate of plasminogen also binds to the VEC surface. Our previous findings 6 showed that fluorescent‐labeled plasminogen progressively accumulated on tPA‐GFP‐expressing VEC surface/pericellular areas in a manner that was tPA‐GFP activity‐dependent and plasmin activity‐dependent. Our prior findings further demonstrated that the surface‐retained tPA‐GFP effectively initiated the dissolution of fibrin clots (fibrinolysis) that had formed over tPA‐GFP‐expressing cells.

The prerequisite function of the solid phase was demonstrated in both of the phenomena described in the previous paragraph as follows: First, these phenomena were strongly attenuated when a mutant tPA‐GFP lacking a heavy chain, which is responsible for the retention on VECs as well as fibrin binding, was expressed. Second, the inhibition of plasminogen binding to either the cell surface or fibrin by a lysine analog completely abolished these phenomena. We also demonstrated that plasminogen activator inhibitor‐1 (PAI‐1), the primary inhibitor of tPA, formed a complex with retained tPA and facilitated its dissociation from secretory granule membranes to the fluid phase, which resulted in the reduction of the amount and activity of tPA on VECs 2. The retention of tPA and its modification by PAI‐1 thus appears to be important factors in the regulation of PA activity on the VEC surface.

In this study we aimed to quantify VEC‐associated plasminogen activator activities by two distinct methods: a modified conventional chromogenic assay for the assessment of plasminogen activation, and a clot lysis assay for the assessment of fibrinolytic activities. A combination of these methods enabled us to define the distinct characteristics of both wild‐type and variant tPA (tenecteplase: TNK [T103N, N117Q, K296A, H297A, R298A, R299A]‐tPA), the latter of which is shown to be associated with better reperfusion and clinical improvement as a thrombolytic drug for patients with stroke compared to wild‐type tPA (alteplase) 7 and with safe and feasible agents for minor stroke with intracranial occlusion 8.

## Methods

### Cell culture and plasmids

Vascular endothelial cells, that is, human umbilical vein endothelial cell‐derived EA.hy926 cells—which were established to retain endothelial cell‐specific functions including their fibrinolytic characteristics—were kindly provided by Dr C.J. Edgell of the University of North Carolina at Chapel Hill 9, 10 and cultured as described 2. Using tPA‐GFP plasmid vector 2 as a template, we generated TNK‐tPA‐GFP and K‐tPA (K296A, H297A, R298A, R299A)‐GFP using the QuickChange II site‐directed mutagenesis kit (Stratagene, La Jolla, CA, USA), repeating the reaction several times to achieve the substitutions. The primer information is shown in the Table 1.

**Table 1:** Sequences of primers for variants of tPA‐GFP plasmid vector

Thr‐to‐Asn at amino acid position 103 (T103N)
Forward 5′‐CAG CTA CAG GGG CAA CTG GAG CAC AGC GG‐3′ Reverse 5′‐CCG CTG TGC TCC AGT TGC CCC TGT AGC TG‐3′
Asn‐to‐Gln at amino acid position 117 (N117Q)
Forward 5′‐GTG CAC CAA CTG GCA GAG CAG CGC GTT GG‐3′ Reverse 5′‐CCA ACG CGC TGC TCT GCC AGT TGG TGC AC‐3′
Lys, His‐to‐Ala, Ala at amino acid position 296–297 (K296A, H297A)
Forward 5′‐GCT GCC ATC TTT GCC GCG GCG AGG AGG TCG CCC GG‐3′ Reverse 5′‐CCG GGC GAC CTC CTC GCC GCG GCA AAG ATG GCA GC‐3′
Arg. Arg‐to‐Ala, Ala at amino acid position 298–299 (R298A, R299A)
Forward 5′‐CCA TCT TTG CCG CGG CGG CGG CGT CGC CCG GAG AGC G‐3′ Reverse 5′‐CGC TCT CCG GGC GAC GCC GCC GCC GCG GCA AAG ATG G‐3′

### Analysis of tPA‐GFP retention on cell surface

Cells cultured on 35‐mm glass‐bottom dishes (Asahi Techno Glass, Tokyo, Japan) were transiently transfected with tPAs‐GFP by lipofection using a commercially available transfection reagent (TransIT‐LT1, MIR2300; Mirus, Madison, WI, USA). After overnight incubation, the cells were washed with HEPES‐buffered solution (HBS; 140 mm NaCl, 5 mm KCl, 1 mm MgCl_2_, 2.5 mm CaCl_2_, 10 mm glucose, and 10 mm HEPES‐NaOH, pH 7.3) and then set on the TIRF microscope stage. Details of the microscope system and method of image capture are explained below. The retained ratio (RR) of tPA‐GFP in individual secretory granules after secretion was determined by recording 3‐min movies captured at 3.2 frames per s under the nonstimulated condition. We fit the region of interest (ROI) fitting to a single granule and calculated the averages of the fluorescence intensity in the ROI during the exocytosis procedure. The fluorescence intensity at 2 and 5 s after the opening of the secretory granules was divided by the maximum intensity and is shown as a percentage (RR‐2s and RR‐5s).

### TIRF microscope system

The optical instrument for visualizing the exocytosis process of tPA‐GFP secretory granules consisted of an Olympus inverted microscope (IX81; Olympus Corp., Tokyo, Japan, 60×/1.45 numeric aperture oil‐immersion objective) equipped with a TIRF unit for generating an evanescent field which enabled us to excite only the fluorophore present in the immediate vicinity of the plasma membrane; an automatic focus device (ZDC2); a digital complementary metal oxide semiconductor (CMOS) camera (ORCA‐Flash4.0, C11440; Hamamatsu Photonics, Hamamatsu, Japan), and a temperature controller to keep the cells at 37 °C on the microscope stage (INUG2‐ONID‐BE; Tokai Hit, Shizuoka, Japan). tPAs‐GFP was excited by a 488‐nm laser with a 1.3 neutral density filter (Edmond Optics, Tokyo, Japan), and emissions were collected through a 520/35‐nm band pass filter (Semrock, Rochester, NY, USA). HCImage software (Hamamatsu Photonics) was used to capture the fluorescence images. The fluorescence intensity of an ROI in individual secretory granules was measured and analyzed on an Aquacosmos imaging station (Hamamatsu Photonics).

### Measurement of clot lysis time, plasmin generation, and GFP concentration

Confluent cells in 96‐well plates were used for transfection with plasmids. After the 24‐h cultured supernatant was collected, the cells were washed with HBS and supplemented with 0.5 μm plasminogen (purified from fresh‐frozen human plasma), 1 U·mL thrombin (Benesis, Osaka, Japan), and 3 μm fibrinogen (Enzyme Research Laboratories, South Bend, IN, USA) to form fibrin clots (final volume 200 μL). The clots were overlaid with liquid paraffin, and the absorbance at 405 nm (A405) of each well was measured every 20 min at 37 °C to monitor the turbidity. The clot lysis time was determined as the time when A405 declined to one‐half of its original value.

Plasmin activity on the cell surface was assessed by a chromogenic assay 11. First, the 24‐h cultured supernatant after plasmid transfection to confluent cells in 96‐well plates was collected, and then the cells were washed with HBS and supplemented with 1 μm plasminogen and 0.4 mm S‐2251 (Chromogenix, Mölndal, Sweden) (final volume 100 μL). The reaction mixture was overlaid with liquid paraffin, and A405 was measured every 10 min at 37 °C. The rate of plasmin generation was determined by the following calculation: A405 [m optical density] divided by the square of the time [h]. The values, normalized to estimate the rate of plasmin generation per 1 ng of GFP (which was calculated based on the GFP concentration in individual supernatants) in the variants are shown as the ratio compared to that in the wild‐type.

The concentrations of tPA‐GFP in the supernatants obtained after 24 h of incubation from the individual wells described above were determined using the GFP ELISA Kit (ab117992, Abcam, Cambridge UK).

### Detection of tPAs‐GFP in supernatant by western blotting of GFP

Culture medium was collected after 24 h of incubation from untransfected or transfected cells with tPAs‐GFP expression vectors using a 6‐well culture plate. Under the nonreduced condition, 15 μL of sample mixed with the sample buffer were separated by 7% SDS/PAGE. Protein bands transferred to a nitrocellulose membrane were incubated with anti‐GFP antibody (Molecular Probes, Eugene, OR, USA), and horseradish peroxidase (HRP)‐conjugated secondary antibodies. Signals were visualized by enhanced chemiluminescence (ECL) western blotting detector reagents (GE Healthcare, Little Chalfont, UK).

### Accumulation of plasminogen on VECs

A trace amount of Alexa Fluor 568‐labeled plasminogen (plg‐568) with 0.5 μm nonlabeled plasminogen in HBS was supplemented to tPAs‐GFP‐expressing cells in 35‐mm glass‐bottom dishes and incubated at 37 °C on the stage of a confocal laser scanning microscope (CLSM; FV1000, Olympus). The accumulation of plg‐568 on and around the surface of individual tPAs‐GFP‐expressing cells was determined as the increase in values in plg‐568 fluorescence intensity for 30 min (dF_30 min_; the ratio of fluorescence intensity at 50 min compared to that at 20 min) using the most basal focal images in which the cells were just adhering to the glass.

### Statistical analysis

Correlation coefficients between the clot lysis time/the rate of plasmin generation and the amounts of GFP secretion were evaluated with Pearson's methods for normally distributed data. The significance of the difference in each parameter was evaluated with either the Williams multiple comparison test, Student's *t‐*test (for normal distribution), or Mann–Whitney's *U*‐test (for non‐normal distribution) as needed.

## Results

### Quantitative evaluation of PA activities

**Figure 1:**
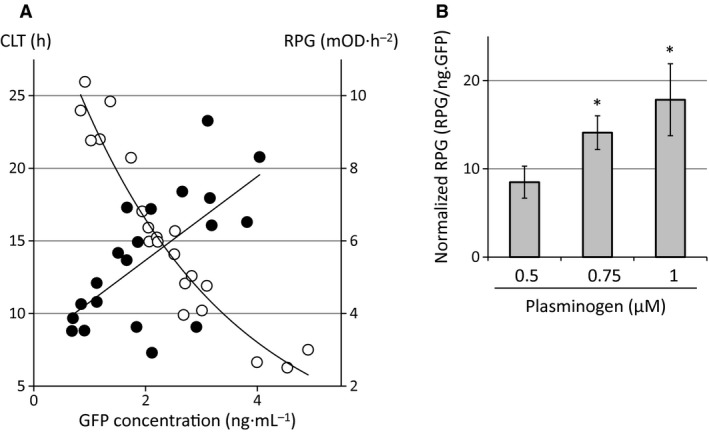
Quantitative evaluation of plasminogen activator activities of expressed tPA‐GFP in EA.hy926 cells. (A) Correlation with GFP concentration in the supernatant after 24‐h transfection from tPA‐GFP‐expressing cells plotted against the clot lysis time (CLT, open circles) or the rate of plasmin generation (RPG, closed circles). The relationship between the GFP concentration and the clot lysis time (*r* = −0.93, *t* = −11.20, *P* < 0.001, *n* = 21) and the rate of plasmin generation (*r* = 0.66, *t* = 3.77, *P* = 0.0014, *n* = 20) can be evaluated by a Pearson's correlation coefficient test. (B) Dose‐dependent effects of supplemented plasminogen. Plasmin generation normalized to a standard unit of 1 ng GFP (RPG/ng.GFP) in supernatant is shown as the mean ± SD (*n* = 6). Statistical significance was determined by a multiple comparison test for parametric data (William's method). *P < 0.01.

Plots of the GFP concentration values in the supernatant from tPA‐GFP‐expressing VECs versus the corresponding clot lysis time (CLT) showed a strong negative correlation (Fig. 1A; open circles, *r* = −0.93, *P* < 0.001). The fibrin clots that formed on the cells not expressing tPA‐GFP showed very little or no dissolution in the 40‐h observation period after clot formation (*n* = 5), indicating that the activity of endogenous plasminogen activators, that is, tPA as well as urokinase‐type plasminogen activator, were not enough to change the fibrin clot turbidity. This inhibition was mainly due to the endogenously expressed PAI‐1, because much of the endogenous tPA existed as tPA‐PAI‐1 complex in the supernatant 2.

The real‐time visualization of fibrin clot lysis using labeled plasminogen and fibrinogen showed that lysis started only where tPA‐GFP‐expressing cells existed 6, suggesting that tPA‐GFP secreted from its expressing cells overcome the inhibitory effect of endogenous PAI‐1 and expresses its plasminogen activator activity at a local area. Therefore, the clot lysis time strongly correlated with the amounts of secreted tPA‐GFP.

**Figure 2:**
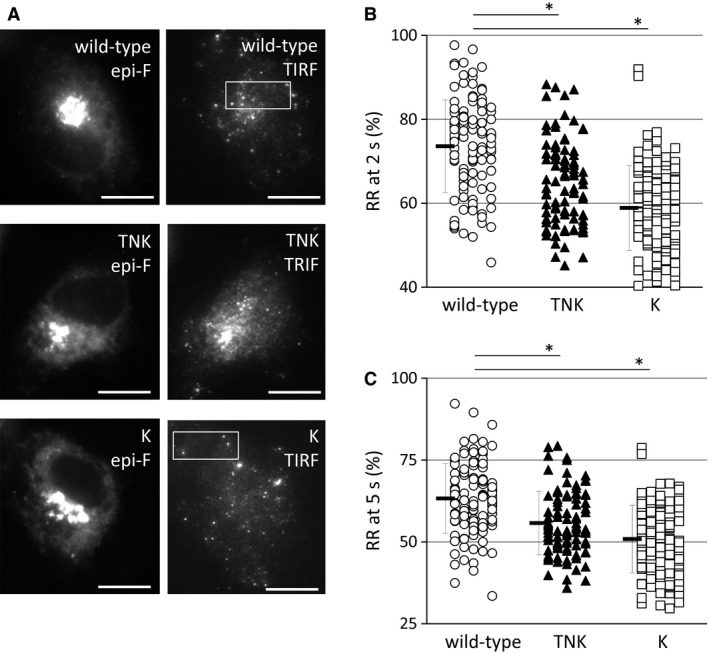
Characteristics of TNK‐/K‐tPA‐GFP‐transfected cells in nonstimulated exocytosis in EA.hy926 cells. (A) Representative images of tPAs‐GFP‐expressing cells. Left panels are epifluorescence (epi‐F) images of wild‐type (upper), TNK‐ (middle), and K‐ (lower) tPA‐GFP‐expressing cells and right are total internal reflection fluorescence (TIRF) images. White boxes in wild‐type and K‐tPA‐GFP images indicate trimming areas as short movie files. Bars: 10 μm. (B, C) The relative amounts of secreted tPAs‐GFP retention at 2 (B) and 5 (C) s after the opening of the secretory granules were estimated as the ratio of the fluorescence intensity at 2 or 5 s as a percentage of the maximum fluorescence intensity. The values of retained ratio at 2 or 5 s (RR at 2‐ or 5‐s) in the wild‐type tPA‐GFP granules (wild‐type, open circles, 117 granules), K‐tPA‐GFP granules (K, open squares, 132 granules), and TNK‐tPA‐GFP granules (TNK, closed triangles, 94 granules) are shown. Means ± SD are indicated by the vertical bars, and Student's *t*‐test was used for the statistical analysis except for TNK in B which showed non‐normal distribution. Mann–Whitney's *U*‐test was applied to TNK in B for statistical analysis. *P < 0.001.

**Figure 3:**
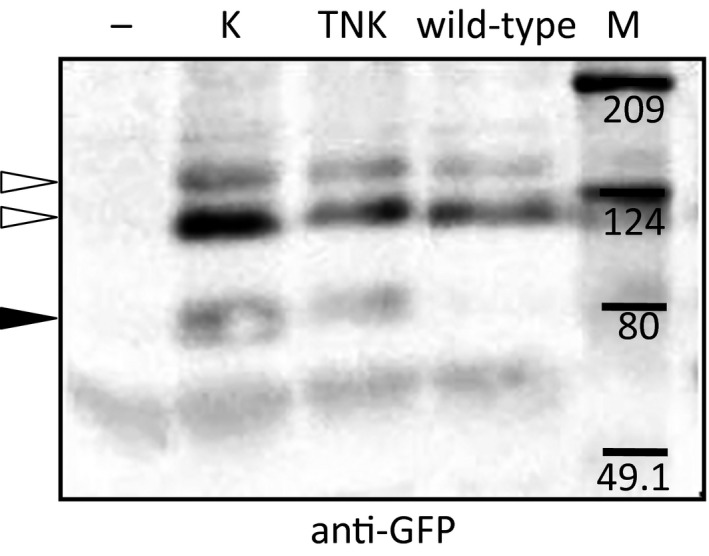
Analysis of supernatant in tPAs‐GFP‐expressing EA.hy926 cells. Western blotting of supernatant obtained after 24‐h incubation from untransfected cells (−) or cells transfected with tPA‐GFP (wild‐type), K‐tPA‐GFP (K), and TNK‐tPA‐GFP (TNK) expression vectors. Protein bands were detected by anti‐GFP antibody. The numbers indicate the position of molecular weight markers (kDa) in lane (M). Open arrowheads: tPAs‐GFP‐PAI‐1 complexes. Closed arrowheads: free tPAs‐GFP.

Similarly, the plots of GFP versus the surface‐associated plasmin generation showed a positive correlation (Fig. 1A, closed circles; *r* = 0.66, *P* = 0.0014). Plasmin generation in cells not expressing tPA‐GFP after 1 μm plasminogen supplementation was detected but was negligible (1.48 ± 0.42 mOD·h^−2^ [mean ± SD, *n* = 5]) even though the cells expressed endogenous plasminogen activators. Plasminogen activation activity, represented as the rate of plasmin generation (RPG)/ng.GFP, normalizing the secretion levels of GFP, was substrate concentration‐sensitive and increased dose‐dependently with increasing concentrations of supplemented plasminogen (Fig. 1B). Although the dissociation rates of tPA‐GFP from the membranous secretory granules after exocytosis were heterogeneous (shown as the retention rate in Fig. 2B, C; open circles), the GFP concentration in the supernatant (mostly tPA‐GFP‐PAI‐1 complex, and a negligibly small amount in free form; Fig. 3, wild‐type) seems to correlate well with the amounts and activities of surface‐retained tPA‐GFP.

### Secretory characteristics of TNK‐/K‐tPA‐GFP

We visualized the secretory dynamics of wild‐type and TNK‐/K‐tPA‐GFP expressed in EA.hy926 cells (Fig. 2A) by using TIRF microscopy. The supplemental movies (Movie S1; wild‐type tPA‐GFP and S2; K‐tPA‐GFP) showed more rapid disperse of exocytosed K‐tPA‐GFP than that of wild‐type tPA‐GFP. We determined the retained ratio (RR) of tPAs‐GFP on the membrane surface after the secretory granular opening, which reflected the residual amounts of tPAs‐GFP on the membrane surface facing the extracellular fluid. The results obtained for TNK‐tPA‐GFP and K‐tPA‐GFP were significantly less than that for wild‐type tPA (Fig. 2B, C), indicating that positively charged amino acid sequences, KHRR(296‐299) residues are important for the retention of tPA‐GFP. In light of our detection of the free forms of TNK‐/K‐tPA‐GFP in 24‐h cultured supernatant, which clearly contrasts with the detection of only the complex form with PAI‐1 in wild‐type tPA‐GFP (Fig. 3), the replacement of KHRR to AAAA seems to have altered the charge‐dependent interaction with the membrane and facilitated the diffusion of TNK‐/K‐tPA‐GFP from the site of the opened granule into the liquid phase. Our trial to confirm the existence of free TNK‐/K‐tPA‐GFP in the supernatant by western blotting using anti‐tPA antibody, however, was not successful due to the fact that the signals derived from the endogenous tPA‐PAI‐1 complex and the expressed free form of tPAs‐GFP were too close and difficult to be distinguished (Data not shown).

### Two differential activities in TNK‐/K‐tPA‐GFP

**Figure 4:**
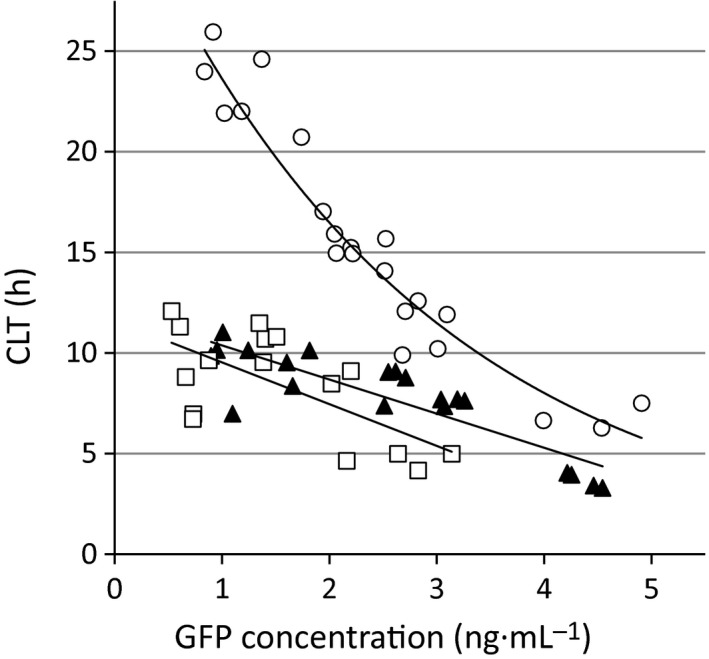
Assessment of clot lysis time triggered by secreted tPAs‐GFP in EA.hy926 cells. GFP concentration in supernatant obtained after 24‐h incubation from tPAs‐GFP‐expressing cells and corresponding values of clot lysis time (CLT) are plotted. Strong correlations of these two factors were obtained using Pearson's correlation coefficient in wild‐type tPA‐GFP (open circles, *r* = −0.93, *t* = −11.20, *P* < 0.001, *n* = 21), in K‐tPA‐GFP (open squares, *r* = −0.67, *t* = −3.34, *P* = 0.005, *n* = 16), and in TNK‐tPA‐GFP (closed triangles, *r* = −0.87, *t* = −7.49, *P* < 0.001, *n* = 20).

We applied our novel quantitative methods to evaluate the activities of tPA variants originated from their unique characteristics. Scatter plots of CLT versus GFP concentrations in 24‐h cultured supernatant from TNK‐/K‐tPA‐GFP‐expressing VECs were largely different and distributed at the left and lower side (Fig. 4, TNK: closed triangles, *r* = −0.87, *P* < 0.001, *n* = 20; and K: open squares, *r* = −0.67, *P* = 0.005, *n* = 16) compared to those from wild‐type tPA‐GFP (Fig. 4, open circles, *r* = −0.93, *P* < 0.001, *n* = 21). This indicated that VEC‐produced TNK‐/K‐tPA‐GFP initiated faster clot lysis than the wild‐type, even though the secretion of TNK‐/K‐tPA‐GFP was lower.

**Figure 5:**
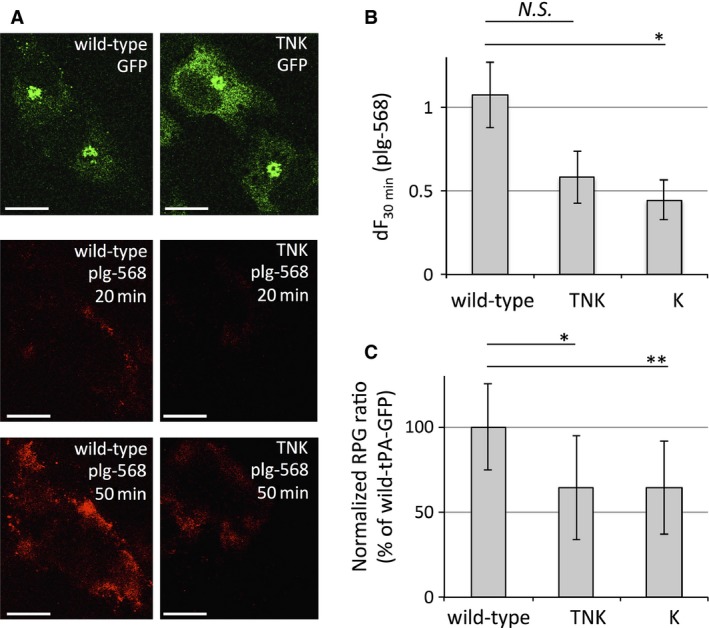
Retained tPAs‐GFP‐triggered plasmin generation in EA.hy926 cells. (A) Representative images of wild‐type (left side) and TNK‐ (right side) tPA‐GFP‐expressing cells. *Upper panels* are fluorescence images of GFP. Fluorescence of plg‐568 was captured every 10 min, and the most basal focal images where cells were just adhering to the glass at 20 min (*middle panels*) and 50 min (*lower panels*) after plg‐568 addition are shown. All images were adjusted with a 50% increase in brightness from the original. Bars: 10 μm. (B) The increasing values in plg‐568 fluorescence intensity for 30 min (the ratio of fluorescence intensity at 50 min compared to that at 20 min) in each tPAs‐GFP‐expressing cell were calculated using an Aquacosmos imaging station and are referred to as dF_30 min_. Bars: mean ± SE of dF_30 min_ from wild‐type (*n* = 13), TNK (*n* = 13), and K (*n* = 8) tPA‐GFP‐expressing cells. (C) The percentages of plasmin generation normalized per unit GFP (RPG/ng.GFP) in TNK‐tPA‐GFP (TNK,* n* = 7), K‐tPA‐GFP (K, *n* = 11) and wild‐type tPA‐GFP (wild‐type, *n* = 14) are shown as mean ± SD. Student's *t*‐test was used; *P < 0.05. **P < 0.005. N.S. indicates *P* = 0.06.

Interestingly, the accumulation of plg‐568 at the cell‐surface/pericellular area where cells were adhering to the glass was significantly decreased in K‐tPA‐GFP (decreased tendency in TNK‐tPA‐GFP; *P* = 0.06)‐expressing cells compared to that in the wild‐type (Fig. 5A,B). This result coincided with the plasminogen activation activity represented as the values of normalized plasmin generation (Fig. 5C), indicating that retained tPA was primarily responsible for the increase in plasmin generation assessed by this method.

## Discussion

Vascular endothelial cells, as well as fibrin clots, provide a solid surface where fibrinolytic components can assemble and interact with each other for effective plasminogen activation 5, 12. We previously demonstrated that the heavy chain of tPA‐GFP is essential for the cell‐surface retention by showing that the heavy‐chain‐deleted mutant form of tPA‐GFP is not retained on the membrane surface after exocytosis 2. Since the retention of tPA‐GFP on a VEC is essential for both cell surface‐associated plasminogen activation and effective fibrin clot lysis 6, we attempted to quantify the expressed tPA‐GFP‐triggered plasminogen activator activities by employing two different methods to analyze fibrinolysis and plasminogen activation. Our present findings demonstrated that the amounts of secreted tPA‐GFP strongly correlated with retained tPA‐GFP‐triggered plasminogen activator activities.

Since most of the tPA‐GFP in the fluid phase exists as a complex form with PAI‐1 in EA.hy926 cells, plasmin generation analysed by a chromogenic assay mainly reflects plasminogen activation initiated by VEC surface‐retained tPA‐GFP. In this facilitated plasminogen activation, augmented plasminogen binding to VEC surface, shown as plg‐568 accumulation in the previous study 6, is indeed deeply involved in the newly exposed C‐terminal lysine residues by the generated plasmin‐dependent degradation of proteins at the cell‐surface/pericellular area.

According to observations of the secretory dynamics of tPA‐GFP by TIRF microscopy, exocytosed tPA‐GFP was retained as fluorescent spots on the membrane surface without any internalization in VECs 2, which is different from that in excitable cells 13, 14. In the present study, the retained fluorescent spots of tPA‐GFP were gradually dimming, suggesting that tPA‐GFP was gradually released. We thus suspected that this slow release of tPA‐GFP consisted of two factors: one is a diffusion of tPA‐GFP from the site of the opened granule into the liquid phase, and the other is a diffusion of tPA‐GFP into the surrounding plasma membrane (lateral diffusion). Although the latter seems more important for the expression of its enzymatic activity, we have not identified the binding molecule of tPA‐GFP on the cell surface, despite our efforts to assess the possible binding to reported tPA receptors 15 such as annexin A2 and galectin 1 (Y. Suzuki, unpubl. data). The fluorescence of tPA‐GFP diffused into the plasma membrane—as well as that on the fibrin clot lytic edge where a large amount of labeled plasminogen accumulated—was less than the detection limit and was hardly visualized 6, but the amount was enough to express plasminogen activator activity. In any case, the GFP concentration in the supernatant, which correlates with the plasminogen activator activity triggered by secreted tPA‐GFP, might be useful as a substitute for the active form of tPA‐GFP. Further, measuring the GFP concentration instead of the tPA concentration to determine the amounts of tPA‐GFP is more practical for assessing variant tPA‐GFP concentrations, since anti‐tPA antibody may react differently with different kinds of variant tPA.

Since PAI‐1 dissociates retained tPA from the VEC surface by forming high molecular weight complexes 2, we expected TNK/K‐tPA‐GFP to be retained longer due to its impaired charge‐dependent interaction with PAI‐1 through its negatively charged residues ‘EEIIMD (350–355)’ 16. Contrary to our expectations, TNK/K‐tPA‐GFP dissociated faster than wild‐type tPA‐GFP after its secretory granular opening. Our present findings demonstrated for the first time that tPA interacts with the plasma membrane surface in a charge‐dependent manner. Further studies are needed to clarify which negatively charged factor is responsible for tPA interactions on the membrane surface.

The reduced binding affinity of TNK/K‐tPA‐GFP toward the membrane developed interesting results in the present novel quantitative methods. In addition to their important characteristics of both higher resistance to PAI‐1 and higher affinity to fibrin clot 17, 18, faster release from VEC surface as a free tPA seems to have also contributed to the more effective fibrin clot lysis. These newly identified characteristics in the present study also contributed toward the less‐effective plasmin generation on the surface of VEC. Thus, the characteristics of tenecteplase having lower affinity to the plasma membrane compared to alteplase might be beneficial as a thrombolytic agent due to the following two reasons. First, higher effective drug concentration in the circulating blood could be expected after intravenous infusion. Second, tPA‐mediated deleterious effects on neurovascular units in the ischemic brain 19, which were partially evoked by the overexpression of VEC surface‐associated plasmin activity 20, 21, could be reduced.

In conclusion, the combination of our novel quantitative methods demonstrated that fibrinolysis and plasmin generation on VECs are distinctly regulated, and the methods were shown to be useful for evaluations of the function of variant tPA molecules with different characteristics. The elucidation of such activity on the surface of VEC, which seems different from those in a purified system, may contribute to the development of a more beneficial tPA variant, including tenecteplase, in which KHRR residues are modified.

## Author contributions

YS designed the study, performed experiments, and wrote the manuscript. HS analyzed and interpreted the data. MT and TB made manuscript revisions. TU supervised the study and wrote the manuscript.

## Supporting information


**Movie S1.** Secretory dynamics of wild‐type tPA‐GFP in EA.hy926. Movie depicting secretory dynamics at partial area indicated as white box in Fig. 2A is shown. Images were acquired at 3.2 frames per s.

**Movie S2.** Secretory dynamics of K‐tPA‐GFP in EA.hy926. Movie depicting secretory dynamics at partial area indicated as white box in Fig. 2A is shown. Images were acquired at 3.2 frames per s.
